# Effect of extracellular vesicles derived from either blood or dental pulp stem cells on human dental pulp stem cells: an in vitro study

**DOI:** 10.1038/s41405-026-00463-8

**Published:** 2026-08-17

**Authors:** Mona A. Amer, Alaa M. Mansour, Mohammed Grawish, Mazen Abou Elkhier

**Affiliations:** 1 Oral Biology department, Faculty of Dentistry, Horus University, New Damietta, Egypt; 2 Oral Biology Department, Faculty of Dentistry Mansoura University, Mansoura, Egypt; 3 Oral Biology Department, Faculty of Oral and Dental Medicine, Delta University for Science and Technology, Dakahlia, Egypt

**Keywords:** Dentistry, Health care

## Abstract

**Objective:**

This study aimed to determine and compare the possible proliferative and differentiative effects of extracellular vesicles (EVs) derived from human dental pulp stem cells (hDPSCs-EVs) and human blood plasma (hBP-EVs) on hDPSCs as an in-vitro assessment for their ability on endogenous hDPSCs for osteo-odontogenic differentiation

**Materials and methods:**

hDPSCs were isolated from impacted third molars of healthy donors and characterized by flow cytometry. EVs were isolated from hDPSCs-conditioned media and human plasma using differential ultracentrifugation and characterized by transmission electron microscopy (TEM), nanoparticle tracking analysis (NTA), bicinchoninic acid assay (BCA), and Western blotting (WB). hDPSCs were cultured in standard medium (control) or supplemented with 50 µg/mL hDPSCs-EVs or hBP-EVs. Proliferation was assessed by MTT assay. Mineralization was evaluated by Alizarin Red staining. Odontogenic differentiation was analyzed by quantitative real-time PCR for dentin sialophosphoprotein (DSPP) expression and immunocytochemical detection of osteocalcin (OCN).

**Results:**

By flow cytometry, hDPSCs showed positive expression for CD90 and CD105 and negative expression for CD34 and CD45. Both EVs types demonstrated characteristic nanoscale morphology and EVs marker expression. hDPSCs-EVs significantly enhanced proliferation compared with hBP-EVs and control (*P* < 0.001). Additionally, mineral deposition, DSPP expression, and OCN-positive area were markedly higher in the hDPSCs-EVs group than in the hBP-EVs and control groups (*P* < 0.001).

**Conclusion:**

Although hBP-EVs exhibited measurable regenerative activity, tissue-specific hDPSCs- EVs produced significantly greater proliferative and osteo-odontogenic effects. These findings suggest that EVs bioactivity is strongly influenced by cellular origin and highlight the importance of lineage-specific vesicles in optimizing cell-free regenerative strategies for endodontic applications

**Figure:**
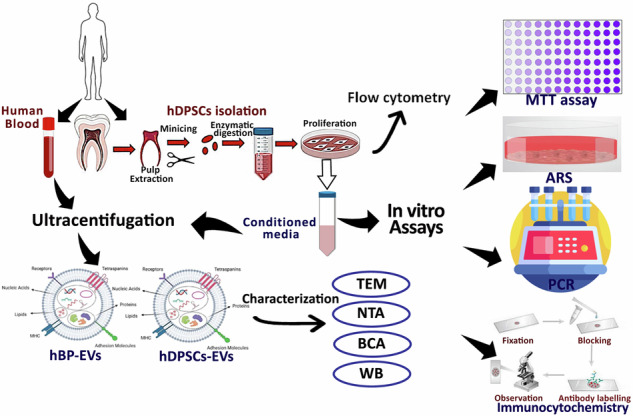

## Introduction

Preservation of pulp vitality is fundamental to long-term tooth survival, as loss of vitality is frequently associated with structural compromise, functional deterioration, and eventual tooth loss. The dentin–pulp complex possesses intrinsic defense and reparative mechanisms that contribute to its resilience. Odontoblasts constitute the first line of defense by producing primary, secondary, and reactionary dentin. When odontoblasts are irreversibly damaged, dental pulp stem cells (DPSCs) can differentiate into odontoblast-like cells and generate reparative dentin, thereby restoring structural integrity and maintaining pulp vitality [1].

Clinically, the pulp may be vital, inflamed, or necrotic. While vital pulp therapies aim to preserve tissue integrity in cases of inflammation, pulpal necrosis necessitates removal of the necrotic tissue and replacement with inert filling materials. Although conventional endodontic treatment effectively controls infection, it results in a non-vital tooth that lacks sensory and defensive functions and may exhibit reduced long-term biomechanical performance. Consequently, biologically driven regenerative endodontic strategies based on tissue engineering principles have emerged as promising alternatives to restore functional pulp tissue rather than merely replacing it [2].

DPSCs are considered a highly promising cell source for dentin–pulp regeneration due to their robust proliferative capacity and multilineage differentiation potential. However, direct stem cell transplantation is associated with potential limitations, including tumorigenicity, immune-related concerns, ethical considerations, and regulatory challenges. Increasing evidence indicates that the regenerative effects of stem cells are largely mediated through paracrine signaling, particularly via EVs (30–150 nm), which function as critical mediators of intercellular communication. Mesenchymal stem cell–derived EVs promote proliferation, migration, angiogenesis, neurogenesis, and odontogenesis, highlighting their potential as cell-free therapeutic agents for dentin–pulp regeneration [3].

Unlike living cells, EVs offer practical advantages, including stability, reduced immunogenicity, and suitability for storage and off-the-shelf application. EVs are present in various biological fluids, including saliva, urine, and plasma. hBP-EVs are primarily released from erythrocytes and platelets, although other circulating cells such as mast cells, neutrophils, and eosinophils also secrete EVs; currently, there is no evidence supporting EVs secretion from basophils [4].

Compared with hDPSCs-EVs, hBP -EVs present distinct translational advantages. Blood collection is minimally invasive, widely accessible, and amenable to repeated sampling, facilitating scalable EVs production. Moreover, hBP-EVs reflect systemic regenerative and immunomodulatory signaling and have demonstrated bioactivity in promoting proliferation, angiogenesis, and tissue repair. Additionally, standardized blood processing protocols further enhance reproducibility and clinical feasibility [5].

However, despite the increasing interest in EVs-based regenerative therapies, direct comparative studies evaluating the biological efficacy of tissue-specific EVs versus systemic plasma-derived EVs in dentin–pulp regeneration remain limited. It remains unclear whether lineage-specific EVs derived from DPSCs provide superior odontogenic signaling compared with the more clinically accessible plasma-derived EVs. This knowledge gap is critical for determining the most effective and translationally feasible EVs source for regenerative endodontic applications.

Therefore, the present study aimed to comparatively evaluate the proliferative and osteo-odontogenic differentiation effects of extracellular vesicles derived from human dental pulp stem cells and human blood plasma on human dental pulp stem cells in vitro. We hypothesized that hDPSCs- EVs would exert stronger proliferative and odontogenic effects than hBP -EVs due to their tissue-specific signaling cargo.

## Materials and methods

### Sample size calculation and power of the study

Sample size calculation was performed using G*POWER statistical software (version 3.1.9.2) for a comparative study between two groups. Based on data derived from previous study [6], who found a significant difference between groups, the calculation revealed that the required sample size for this study was 16 patients per group. Calculations were made using *α* = 0.05, power 80% and effect size = 1.05 and allocation ratio N2/N1 = 1.

### Samples collection of teeth and plasma

Teeth and blood samples were collected according to strict inclusion and exclusion criteria. *For teeth selection*, inclusion criteria comprised impacted, caries-free third molars obtained from healthy, non-smoking, non-alcohol-consuming males aged 20–35 years. Teeth showing decay, infection, exposed pulp, or root resorption were excluded. *For blood sample collection*, donors were healthy, non-smoking, non-alcohol-consuming males within the same age range. Samples from donors outside this age range, or those exhibiting hemolysis, improper storage, infections, or chronic diseases, were excluded. All samples were non-hemolyzed and either processed immediately or stored at −80 °C until use. Written informed consent and relevant health documentation were obtained from all donors.

### Isolation and characterization of hDPSCs

Teeth were irrigated with normal saline, 70% ethyl alcohol, and PBS, then preserved in pasteurized milk. Pulp was extracted using a handheld pulp isolator device (WO2016162041A9) and minced. Tissue was digested with collagenase type I (3 mg/mL) and dispase type II at 37 °C, strained, centrifuged, and cultured in Dulbecco’s Modified Eagle Medium- F12 (DMEM-F12) with 10% FBS (SigmaAldrich) and 1% penicillin/streptomycin (Pen Strip, 100 ml, gibco, USA), at 37 °C, 5% CO₂. For subculturing, cells were trypsinized at 80% confluence using 0.25% Trypsin/EDTA; third-passage cells were used.

The phenotypic characterization of hDPSCs was carried out through flow cytometry analysis. Immunophenotyping employed antibodies targeting human antigens, with CD90 and CD105 used as positive markers, whereas CD34 and CD45 (BD Biosciences, USA) were used as negative markers. A total of 1 × 10^6^ cells were resuspended in a flow cytometry staining buffer. It was performed using CytoFLEX S Flow Cytometer (Beckman Coulter, USA) and CytExpert software [7]. To evaluate the cells’ differentiation, the cells were incubated with either complete culture media or osteogenic media for 14 days. The osteogenic media consisted of culture media supplemented with 10 mM β-glycerol phosphate, 100 nM dexamethasone, and 50 mg/mL ascorbic acid [8].

### Extracellular vesicles isolation and characterization

For hDPSCs- EVs, conditioned media from 70–80% confluent hDPSCs were centrifuged sequentially: 300 × *g* (10 min), 2000 × *g* (20 min), 10,000 × *g* (30 min), and 100,000 × *g* (60 min) at 4 °C. Pellets were washed and resuspended in PBS. For hBP-EVs isolation, 50 mL blood in EDTA tubes was centrifuged at 900 × *g* for 20 min, then 4500 × *g* for 10 min. Plasma was diluted 1:10 with 0.9% NaCl, filtered (0.22 μm), and ultra-centrifuged at 100,000 × *g* for 4 h at 4 °C [9] Due to the higher protein complexity and lower vesicle concentration in plasma compared with conditioned media, plasma samples were ultra-centrifuged for 4 h at 100,000 × *g* to enhance EVs recovery efficiency, whereas conditioned media–derived EVs were pelleted using a standard 60-min ultracentrifugation protocols [10, 11] EVs were characterized utilizing transmission electron microscopy (TEM), nanoparticle tracking analysis (NTA), the bicinchoninic acid (BCA) assay and western blot analysis (WB).

### Biophysical characterization of extracellular vesicles

The pellets obtained from ultracentrifugation at 100,000 × *g* were fixed in 2% paraformaldehyde (PFA), stained with uranyl-oxalate (pH 7) and methyl cellulose-UA. The EVs were examined using TEM (JEOL JEM-2100) operating at 160 kV at the Electron Microscope Unit, Mansoura University, Egypt. The size distribution and concentration of hDPSCs-EVs as well as hBP-EVs were assessed using a particle matrix device for NTA (ZetaView® x30, Particle Metrix GmbH, Germany). To ensure the EVs concentration fell within the device’s optimal detection range, approximately 10⁸ particles/mL, samples were diluted in sterile PBS [12].

### Bicinchoninic acid assay

The bicinchoninic acid (BCA) protein assay kit (Cat. no. 23227, Thermo Scientific, Rockford, IL, US) was used to quantify protein levels, with bovine serum albumin (BSA, Cat. no. A4503, Sigma Aldrich, US) serving as the standard. For the BCA assay, 25 µL of each protein standard and sample replicate was pipetted into individual wells of a 96-well microplate. Subsequently, 200 µL of the freshly prepared working reagent (WR) was added to each well. The WR was prepared immediately before use by mixing Reagent A, containing bicinchoninic acid, sodium carbonate, sodium bicarbonate, sodium tartrate, and sodium hydroxide, with Reagent B, containing copper (II) sulfate pentahydrate, in a 50:1 volumetric ratio. Under alkaline conditions, proteins in the samples reduce Cu²⁺ ions from Reagent B to Cu⁺, which then form a stable purple-colored chelate complex with bicinchoninic acid. To ensure thorough mixing, the plate was placed on a shaker for 30 seconds. After mixing, the microplate was covered and incubated at 37 °C for 30 min to allow the colorimetric reaction to develop, indicating protein presence. The intensity of the resulting color, measurable at 562 nm, is directly proportional to the protein concentration in the sample [13].

### Western blot analysis

The EVs’ protein content was detected using Western blot (WB). EV pellets were lysed using protease cocktail inhibitors and Radioimmunoprecipitation assay buffer (1x RIPA) to extract their proteins. The Bradford assay was utilized to assess the total protein content. The extracted proteins were dissolved in Laemmli buffer to enable separation by size using SDS-PAGE. During electrophoresis, an electric current was applied to separate the proteins based on molecular weight. Following separation, the proteins were moved from the gel to a PVDF membrane utilizing the “transfer sandwich” method on a BioRad Semi-Dry Blotter. To detect specific EVs proteins, the membrane underwent sequential incubation steps. First, it was blocked for 1 h in 5% milk in TBS-Tween to reduce non-specific antibody binding and enhance the signal-to-noise ratio. Then the membrane was incubated with primary antibodies CD63, CD81, CD9 and syntenin diluted 1:1000 in the blocking solution overnight at 4 °C. After washing off unbound primary antibodies, the membrane was incubated with horseradish peroxidase (HRP)-conjugated secondary antibodies (sheep anti-mouse or mouse anti-goat) at a 1:10,000 dilution in the blocking solution. For detection, the membrane was treated with ECL™ Prime Western blotting detection reagents for 5 min. The reagents interacted with the HRP-conjugated secondary antibodies, producing chemiluminescence. The resulting protein bands were visualized using the Intas Chemostar Plus imaging system with ChemoStar software v.0.5.67 [14].

### In vitro assays

Different in-vitro assays were performed to evaluate the effect of hDPSCs-EVs and hBP-EVs on hDPSCs behavior in proliferation, and osteo/odontogenic differentiation. Protein-based EVs quantification remains one of the most widely utilized approaches for EVs dosing standardization in in vitro functional studies [15] Therefore, EVs concentration in this study was standardized according to total EVs-associated protein concentration, quantified using BCA assay, and expressed as µg EVs protein/mL. The selected concentration of EVs in this study was 50 µg/mL depending on a previous article [16], in addition to, other published article that evaluated different EVs concentrations through different assays such as cell proliferation, scratch and transwell assays, where 50 µg/mL showed mostly better results through all assays [17] We also confirmed the EVs concentration in our previous article as we evaluated different EVs concentrations on dental pulp cells’ viability and 50 µg/mL showed better results as well [18].

For cell viability assay; methyl-tetrazolium assay *(MTT Assay)*, hDPSCs were divided into three groups. Group I (control group): hDPSCs cultured in complete culture media, group II: hDPSCs cultured in complete culture media containing 50 µg/mL hDPSCs-EVs, and group III: hDPSCs cultured in complete culture media containing 50 µg/mL hBP-EVs. 5 × 10^3^ cells of hDPSCs at passage three were seeded in each well of 96-well plates. Complete culture medium alone, or either containing hDPSCs- EVs or hBP-EVs, was added, then the plates were incubated in the CO_2_ incubator. After 1, 3 and 5 days, 12 mM of MTT solution (5 mg/mL) was utilized to substitute the culture medium. Then, the wells were incubated for 4 h. After 4 h, the MTT solution was discarded and 150 µL of dimethyl sulfoxide was added to each well to dissolve the formazan crystals. Finally, absorbance level was measured at 570 nm via a microplate reader (BioTek ELx 800) (HIGHLAN PARK, USA) Cat. No. 269050. The assay was repeated at least 3 times.

For odontogenic/osteogenic differentiation assay, *mineralization matrix deposition assay, and odontogenic gene expression*. hDPSCs were divided into four groups. Group I (negative control): hDPSCs cultured in complete culture media, group II (positive control): hDPSCs cultured in osteogenic medium which comprised 10 mM β-glycerophosphate, 50 µg/mL ascorbic acid, 10 nM dexamethasone, group III (hDPSCs-EVs group): hDPSCs cultured in osteogenic medium containing 50 µg/mL hDPSCs-EVs and group IV (hBP-EVs group): hDPSCs cultured in osteogenic medium containing 50 µg/mL hBP-EVs. 6-well plates were used with a cell density of 5 × 10^4^ for each well. All groups were incubated at 37 °C and 5% CO_2_. Cells were daily examined with the aid of an inverted light microscope (Olympus Optical Co., Ltd., Japan). Every 3 days, the osteogenic media were changed, and the test was repeated at least three times for each group. After 14 days, hDPSCs were fixed for 15 min in 10% (v/v) formaldehyde, stained with 250 μl of 40 mM Alizarin Red staining (ARS, pH 4.1). ARS was used to assess the formation of mineralized nodules. For quantification, the stained cells were dissolved in 10% acetic acid and quantified using a microplate reader Azure Ao absorbance microplate reader (Azure Biosystems, USA) at 405 nm.

### Real-time polymerase chain reaction (qRT-PCR) analysis

The hDPSCs in different groups were cultured and incubated within the Greiner flask with 2.5 × 10^4^ cell density. After 14 days, hDPSCs of each group were harvested by trypsinization, transferred to 15 ml sterile falcon tube, then the total RNA was extracted and purified directly by the RNeasy® Mini Kit [50] (LOT #: 163046464, QIAGEN GmbH, Hilden, Germany) based on the manufacturer’s recommendations [19]. The total RNA was later subjected for reverse transcription utilizing the Revert Aid First Strand cDNA Synthesis Kit (LOT #: 00886149, Thermo Fisher Scientific, Baltic, Lithuania) in accordance with the manufacture’s guidelines [20]. The method of reverse transcription is used to create complementary DNA (cDNA). Unlike mRNA, cDNA is extremely stable and can be quantified using PCR. The levels of mRNA expression of the dentin sialo phosphoprotein (DSPP), were measured in cultured hDPSCs for the four different groups and for fold change.

Beta-actin (β-actin) was measured using the designed primers originated utilizing Basic Local Alignment Tool (BLAST software, https://blast.ncbi.nlm.nih.gov/Blast.cgi). The cDNA was employed as a template for preferred gene amplification with particular primers by real-time PCR using Thermo Scientific™ ARKTIK™ Thermal Cycler Type 5020 96-well Single Block (Thermo Fisher Scientific, Vantaa, Finland). The PCR was carried out using the Thermo Scientific PikoReal Real-time PCR System (Thermo Fisher Scientific, Vantaa, Finland) with the aid of SYBR green. The list of forwarding and reverse primers of selected genes was displayed in (Table 1) [19, 20] The reaction’s protocol included initial denaturation step at 95 °C for 2–3 min, then denaturation step at 95 °C for 10 min, after that annealing at 55–65 °C for 20–30 s and a final extension step at 72 °C for 20–30 s as demonstrated in (Table 2).

**Table 1:** The primers used for **qRT-PCR** hDPSCs as well as the list of forwarding and reverse primers of selected genes.

mRNA Symbol	Full name	Accession number	Primer sequence	Chromosome location	Ensemble number
DSPP	Dentin Sialo Phosphoprotein	NM_014208.3	5-TTAAATGCCAGTGGAACCAT-3 5-ATTCCCTTCTCCCTTGTGAC-3	4q22.1	ENSG00000152591
β-actin	Beta-actin	X00351.1	5-CTCTTCCAGCCTTCCTTCCT-3 5- AGCACTGTGTTGGCGTACAG-3	7p22.1	ENSG00000075624

**Table 2:** The temperature, time and number of cycles for each step.

Process	Temp. (^o^C)	Time	Cycles No.
Initial denaturation	95	2–3 min	1
Denaturation	95	10–15 s	40
Annealing	55–65	30 s	
Extension	72	30 s	

To determine the differences in the amount of cDNA for each gene and to reduce variation caused by PCR protocol and the cycling process, reference genes have been used to normalize the PCRs. Housekeeping genes, such as Beta-actin (β-actin) are commonly used as a reference gene because their expression levels remain stable under different circumstances.

The cycle at which the ΔRn value surpasses the threshold is termed the CT (threshold cycle) representing the number of cycles necessary for the fluorescent signal to exceed the background level. To detect the relative fold changes in gene expression quantitatively, the comparative CT (ΔΔCT) method (Livak method) was utilized as follows: The software measured amplification or the expression levels (represented as the CT values) of the reference & target genes in both treated and control samples. For each reaction, the CT value of each target gene was normalized to the CT value of the housekeeping gene (β-actin gene, which is not changed by any applied treatment), yielding the ΔCT value. The ΔCT of the treated samples was normalized to the ΔCT of the control sample, yielding the ΔΔCT value. Finally, the target gene fold difference in expression between all samples was calculated using the formula 2-ΔΔCT [21].

### Immunocytochemistry

In our study, the Monoclonal Human/Rat Osteocalcin Antibody Mouse IgG1Clone # 190125 was used with Catalog Number: MAB1419 and dilution of 1:100. It was supplied by Bio-Techne, Minneapolis, USA. For immunocytochemical Staining for Osteocalcin: cells were fixed with 4% paraformaldehyde, permeabilized with 0.1% Triton X-100, and blocked with 5% BSA to prevent non-specific binding. They were then incubated overnight at 4 °C with the primary antibody (clone OCN-1, 1:100 dilution), followed by washing and incubation with an HRP-conjugated anti-mouse IgG secondary antibody (1:500, Thermo Fisher) for 45 min at room temperature. Detection was carried out using DAB substrate, producing a brown precipitate at osteocalcin-positive sites, after which the reaction was terminated by washing with distilled water. After that, Cells were counterstained with hematoxylin (1–2 min) to visualize nuclei. Coverslips were rinsed in water, dehydrated through graded ethanol (70%, 95%, 100%) and cleared in xylene. Coverslips were then mounted on glass slides using permanent mounting medium (e.g., DPX, Thermo Fisher) and cells were ready for microscopy and imaging [22].

### Quantification of osteocalcin immunostaining

Images of osteocalcin-immunostained cells were captured under consistent magnification and exposure using a brightfield microscope. High-resolution TIFF images were analyzed with ImageJ (version X.X; NIH, USA). Images were converted to 8-bit grayscale and contrast-adjusted uniformly. Osteocalcin-positive regions were delineated via thresholding and converted to binary masks. The Analyze Particles function quantified the stained area, and percentage area was calculated as:

$${{\rm{Percentage}}}\; {{\rm{Area}}}={{\rm{Osteocalcin}}}-{{\rm{positive}}}\; {{\rm{areas}}}\times 100/ {{\rm{total}}}\; {{\rm{image}}}\; {{\rm{area}}}$$

Percentage area measurement was chosen as it provides an objective, reproducible estimate of protein expression across the entire field of view. All images were analyzed in triplicate. For variable staining intensity, DAB-specific color deconvolution was applied prior to thresholding.

### Statistical analysis

Data were expressed as mean ± standard deviation (SD). ANOVA was employed to assess the overall significance among greater than two groups, succeeded by Tukey’s honest significant difference post hoc test for pairwise comparisons. The significance level was determined at *P* ≤ 0.05. Statistical analysis was conducted with IBM SPSS Statistics for Windows, version 23.0.

### Ethical approval

This research was accepted by the Ethical Committee of the Faculty of Dentistry, Mansoura University, Egypt, code number: A01011122. Informed consents were acquired from all participants informing them about the purpose and the possible outcomes of the research. The procedures used in this research adhered to the ethical guidelines defined in the Declaration of Helsinki. All experimental work, including the culture and differentiation of human dental pulp stem cells and other procedures, was conducted at the Mansoura Experimental Research Center *(MERC)*, Mansoura University. The study followed institutional ethical guidelines and international standards to ensure proper handling of biological materials and adherence to ethical research practices.

## Results

### Characterization of hDPSCs

**Fig. 1:**
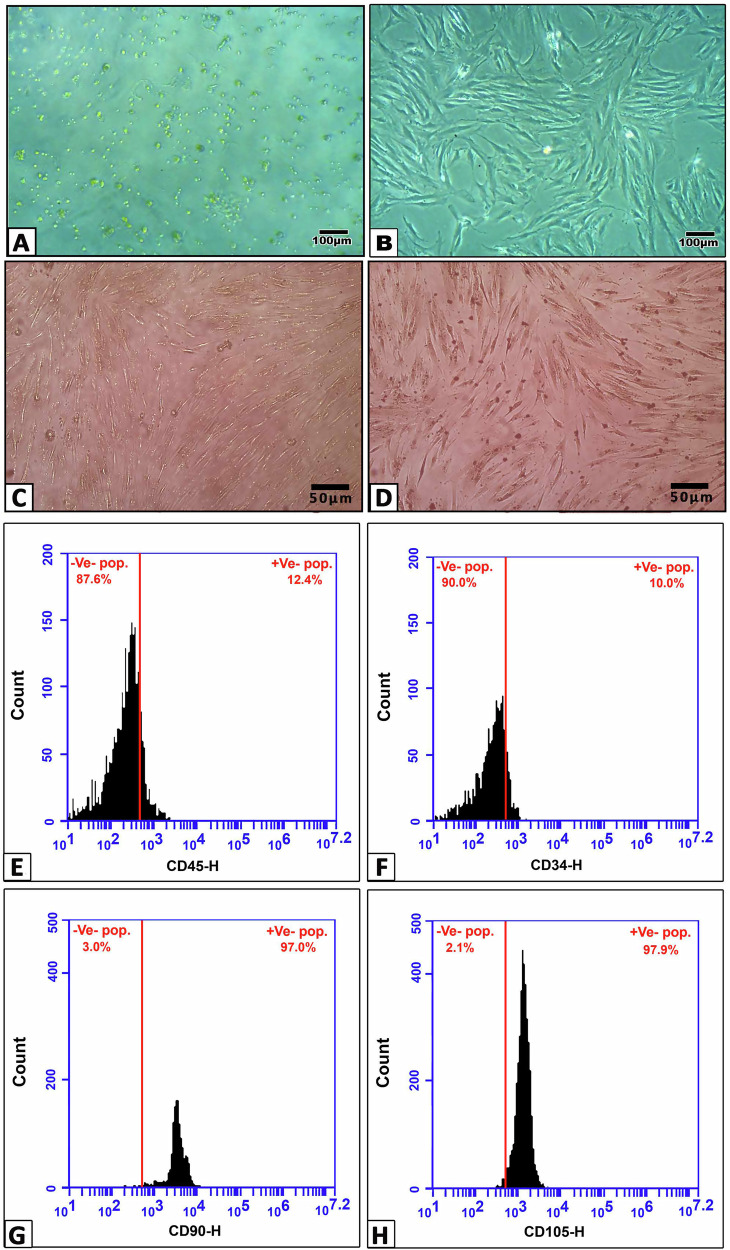
Characterization of human dental pulp stem cells (hDPSCs). Inverted light microscopic photomicrographs demonstrating (**A**) isolated hDPSCs exhibiting an initially rounded morphology, (**B**) colony formation capacity of hDPSCs by spindle-shaped cells. Osteodifferentiation of hDPSCs showing (**C**) cells cultured in complete culture medium, and (**D**) osteogenic differentiation of hDPSCs following culture in osteogenic induction medium. Flow cytometry histograms showing the expression of (**E**) CD45 (12.4% positivity), (**F**) CD34 (10.0% positivity), (**G**) CD90 (97.0% positivity), and (**H**) CD105 (97.9% positivity).

Under inverted light microscopy, the isolated cells were monitored daily until subculturing. Initially, cells appeared rounded and non-adherent. After 24 h., part of the cells transitioned to a fibroblastic spindle-like morphology and attached to the flask. By day 7, there was a notable increase in adherent cells. Cell colonies became visible and increased in size day by day. Subculturing was done when the cells reached confluence, forming a monolayer of spindle-shaped cells (Fig. 1A, B). Additionally, the cells differentiation was confirmed through Osteodifferentiation by presence of calcified nodules especially with osteogenic media (Fig. 1C, D). Via flow cytometry, immunophenotypic characterization confirmed the mesenchymal stromal/stem cell–like phenotype of the isolated hDPSCs. The cells were negative for hematopoietic markers CD45 (12.4% positivity) and CD34 (10% positivity), while exhibiting high expression of MSCs markers CD90 (97% positivity) and CD105 (97.9% positivity). These results demonstrate a high yield and purity of hDPSCs isolated from human dental pulp tissue. (Fig. 1E, F, G, H).

### Characterization of EVs

**Fig. 2:**
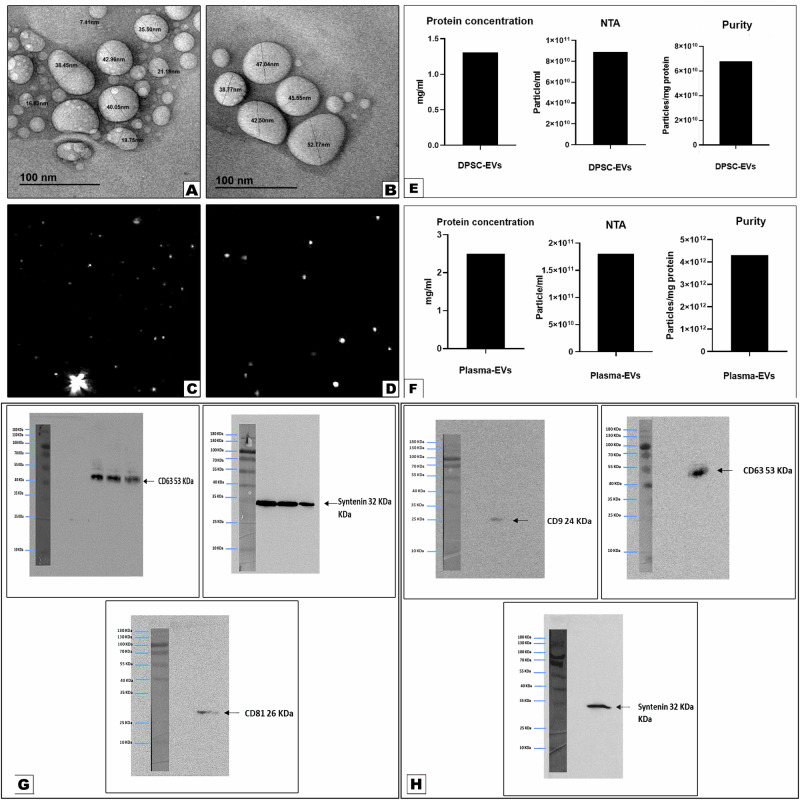
Characterization of human dental pulp stem cell-derived extracellular vesicles (hDPSCs-EVs) and human blood plasma-derived extracellular vesicles (hBP-EVs). Transmission electron microscopy (TEM), nanoparticle tracking analysis (NTA), particle-to-protein ratio analysis, and Western blot characterization of hDPSCs-EVs and hBP-EVs. (**A**, **B**) TEM micrographs demonstrating the characteristic spherical/cup-shaped morphology of hDPSCs-EVs and hBP-EVs, respectively. (**C**, **D**) NTA profiles showing the size distribution and particle concentration of hDPSCs-EVs and hBP-EVs, respectively. (**E**, **F**) Particle-to-protein ratio analysis used to assess the purity of hDPSCs-EVs and hBP-EVs, respectively. (**G**, **H**) Western blot analysis confirming the expression of the EVs-associated markers CD9, CD63, CD81, and Syntenin in hDPSCs-EVs and hBP-EVs, respectively.

Both hDPSCs-EVs and hBP-EVs were isolated using differential ultracentrifugation. Under TEM, it revealed that both types of EVs possessed a spherical or cup-plate-shaped morphology, with diameters in the nanoscale range (100 nm scale), consistent with EV characteristics. (Fig. 2A, B). NTA was performed to evaluate EV particle size distribution and concentration. The analysis demonstrated that hDPSCs-EVs had a median diameter (*X*₅₀) of 133.2 nm and a concentration of 8.9 × 10¹⁰ particles/cm³, with consistent size distribution peaking at 133.2 nm (Fig. 2C), while hBP-EVs had a median diameter of 117.6 nm and a higher concentration of 1.8 × 10¹¹ particles/cm³, also with uniform distribution (Fig. 2D).

Protein concentration, as quantified by BCA assay, was 1.308 mg/mL for hDPSCs-EVs and 2.505 mg/mL for hBP-EVs. EVs purity was also calculated as the ratio of particle concentration (from NTA) to protein concentration (from BCA). hDPSCs-EVs exhibited a purity of 6.8 × 10¹⁰ particles/mg, (Fig. 2E). while hBP-EVs had a higher purity of 4.31 × 10¹² particles/mg. (Fig. 2F). Using Western blot analysis for protein identification, the presence of EVs markers was verified. hDPSCs-EVs expressed CD63 (~53 kDa), CD81, and syntenin, with robust bands for CD81 and syntenin, though CD63 detection suggested potential for optimization (Fig. 2G). hBP-EVs expressed CD9, CD63, and syntenin, confirming successful EV isolation (Fig. 2H).

### In vitro assays

#### Cell proliferation (MTT assay)

The effect of EVs on hDPSCs proliferation was evaluated using the MTT assay over 1, 3, and 5 days. On day 1, mean optical density (OD) values were 0.174 ± 0.002 (control), 0.287 ± 0.012 (hDPSCs-EVs), and 0.185 ± 0.001 (hBP-EVs). One-way ANOVA indicated significant differences among groups (*P* < 0.001), with Tukey’s post hoc test showing significance between control and hDPSCs-EVs (*p*1 < 0.001), hDPSCs-EVs and hBP-EVs (*p*3 < 0.001), but not between control and hBP-EVs (*p*2 = 0.195).

**Fig. 3:**
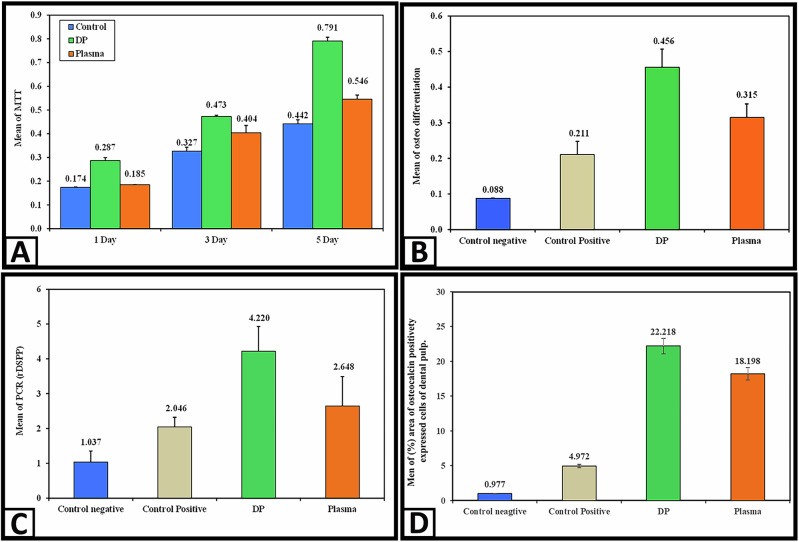
Quantitative analysis of hDPSCs-EVs and hBP-EVs effects on hDPSCs proliferation and osteogenic differentiation. Bar charts representing (**A**) the mean values of the MTT assay for cell viability/proliferation among the different groups after 1, 3, and 5 days; (**B**) the mean values of the Alizarin Red S staining assay for mineralized nodule formation among the different groups; (**C**) relative DSPP gene expression in the tested groups determined by quantitative real-time polymerase chain reaction (qRT-PCR); and (**D**) comparison of the percentage area (%) of osteocalcin (OCN)-positive immunocytochemical staining among the different study groups.

On day 3, OD values increased to 0.327 ± 0.016 (control), 0.473 ± 0.005 (hDPSCs-EVs), and 0.404 ± 0.031 (hBP-EVs). ANOVA confirmed group differences (*P* < 0.001), with all pairwise comparisons significant (*p*1 < 0.001, *p*2 = 0.008, p3 = 0.013). On day 5, OD values were 0.442 ± 0.017 (control), 0.791 ± 0.016 (hDPSCs-EVs), and 0.546 ± 0.017 (hBP-EVs). Again, ANOVA showed significance (*P* < 0.001), with all pairwise differences notable (*p*1 < 0.001, *p*2 = 0.001, *p*3 < 0.001). Proliferation was highest on day 5 across all groups, with hDPSCs-EVs demonstrating superior proliferative stimulation compared to hBP-EVs and control (Table 3; Fig. 3A).

**Table 3:** Results of MTT assay and their statistical significance for 50 μg/mL concentrations of extracellular vesicles (EVs) among studied groups after 1, 3 and 5 days.

period	MTT	I (Control) (*n *= 16)	II (hDPSCs-EVs) (*n* = 16)	III (hBP-EVs) (*n* = 16)	*P* value
1 Day	Mean ± SD.	0.174 ± 0.002	0.287 ± 0.012	0.185 ± 0.001	<0.001^*^
	Sig. bet. grps.	*p*_1_ < 0.001^*^, *p*_2_ = 0.195, *p*_*3*_ < 0.001^*^			
3 Day	Mean ± SD.	0.327 ± 0.016	0.473 ± 0.005	0.404 ± 0.031	<0.001^*^
	Sig. bet. grps.	*p*_1_ < 0.001^*^, *p*_2_ = 0.008^*^, p_3_ = 0.013^*^			
5 Day	Mean ± SD.	0.442 ± 0.017	0.791 ± 0.016	0.546 ± 0.017	<0.001^*^
	Sig. bet. grps.	*p*_1_ < 0.001^*^, *p*_2_ = 0.001^*^, *p*_3_ < 0.001^*^			

Data was expressed using Mean ± SD.SD: **Standard deviation**. *p* value for comparing between the three studied groupsp_1_*: p* value for comparing between **Control** and **DP** *p*_*2*_*: p* value for comparing between **Control** and **Plasma**p_3_: *p* value for comparing between **DP** and Plasma*: Statistically significant at *p* ≤ 0.05

### Osteogenic/odontogenic differentiation

#### Alizarin Red staining

**Fig. 4:**
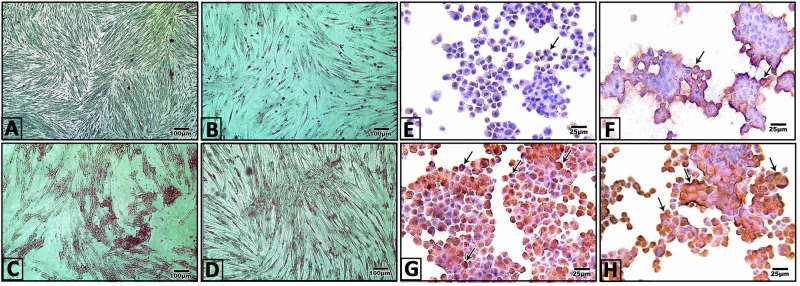
Representative images of mineralization and osteocalcin (OCN) expression in hDPSCs following treatment. Inverted light microscopic photomicrographs at 100× magnification showing Alizarin Red S-stained mineralized nodule formation in hDPSCs in the control negative group (**A**), control positive group (**B**), test group 1 (**C**), and test group 2 (**D**). Scale bar = 100 μm. Inverted light microscopic photomicrographs at 40× magnification showing OCN immunocytochemical staining in hDPSCs in the control negative group (**E**), control positive group (**F**), test group 1 (**G**), and test group 2 (**H**). Positive OCN expression is indicated by brown staining. Scale bar = 25 μm.

It revealed mineralization nodules after 14 days. The group I (negative control) showed minimal, red-stained calcification areas with trace calcium deposits (OD: 0.088 ± 0.001). (Fig. 4A). The group II (positive control) displayed slightly larger calcified areas with scattered deposits (OD: 0.211 ± 0.037). (Fig. 4B). Group III (hDPSCs-EVs) exhibited the largest red areas with multiple large calcium deposits (OD: 0.456 ± 0.051). (Fig. 4C), while test group IV (hBP-EVs) showed intermediate calcification (OD: 0.315 ± 0.038). (Fig. 4D). ANOVA indicated overall significance (*P* < 0.001), with Tukey’s test confirming pairwise differences (*p*1 < 0.001, *p*2 = 0.035, *p*3 = 0.006). Comparisons to negative control were significant for all other groups (p0 = 0.014 for positive control, <0.001 for test groups 1 and 2) (Table 4; Fig. 3B).

**Table 4:** Results of osteodifferentation through mineralization deposition as OD via ARS, and the 2^−ΔΔCt (fold of change) for DSPP relative to β-actin via qRT-PCR, and % area for OCN via ICC, and their statistical significance among different groups.

		I (Control negative) (*n* = 16)	II (Control Positive) (*n* = 16)	III (hDPSCs-EVs) (*n* = 16)	IV (hBP-EVs) (*n* = 16)	*P*
ARS	Mean ± SD.	0.088 ± 0.001	0.211 ± 0.037	0.456 ± 0.051	0.315 ± 0.038	<0.001^*^
	***p***_**0**_		0.014^*^	<0.001^*^	<0.001^*^	
Sig. bet. Groups.		*p*_1_ < 0.001^*^, *p*_2_ = 0.035^*^, *p*_3_ = 0.006^*^				
DSPP	Mean ± SD.	1.037 ± 0.318	2.046 ± 0.277	4.220 ± 0.713	2.648 ± 0.844	0.001^*^
	***p***_***0***_		0.235	0.001^*^	0.041^*^	
Sig. bet. Groups.		*p*_1_ = 0.009^*^, *p*_2_ = 0.617, *p*_*3*_ = 0.046^*^				
OCN	Mean ± SD.	0.997 ± 0.097	4.972 ± 0.853	22.218 ± 1.614	18.198 ± 0.701	<0.001*
	***p*0**		0.005*	<0.001*	<0.001*	
Sig. bet. Groups		*p1* < 0.001*, *p*2 = 0.001*, *p3* = 0.004*				

Data were expressed using Mean ± SD. SD: Standard deviation*: p* value for comparing between the different studied groups *p*_*0*_*: p* value for comparing between Control negative and each other group p_1_: *p* value for comparing between Control Positive and DP *p*_*2*_*: p* value for comparing between Control Positive and Plasma *p*_*3*_*: p* value for comparing between DP and Plasma*: Statistically significant at *p* ≤ 0.05.

### Real-time polymerase chain reaction (qRT-PCR)

Quantified DSPP gene expression after 14 days, normalized to β-actin using the 2^(−ΔΔCt) method. Fold changes were 1.037 ± 0.318 (negative control), 2.046 ± 0.277 (positive control), 4.220 ± 0.713 (test group 1), and 2.648 ± 0.844 (test group 2). ANOVA showed significance (*P* = 0.001), with Tukey’s test indicating differences between positive control and test group 1 (*p*1 = 0.009), test group 1 and test group 2 (*p*3 = 0.046), but not positive control and test group 2 (*p*2 = 0.617). Compared to negative control, significance was observed for test groups 1 (*p*0 = 0.001) and 2 (p0 = 0.041), but not positive control (*p*0 = 0.235) (Table 4; Fig. 3C).

### Immunocytochemistry for OCN

It was performed after 14 days. Positive brown DAB staining was absent in group I (negative control). (Fig. 4E). The positive expression appeared in group II (Fig. 4F) and the expression increased in group III (Fig. 4G) and IV (Fig. 4H). Quantitative analysis of DAB-positive areas yielded percentages of 0.997 ± 0.097, 4.972 ± 0.853, 22.218 ± 1.614, and 18.198 ± 0.701 for group I, II, III and IV, respectively. ANOVA confirmed significance (*P* < 0.001), with all pairwise comparisons significant (p1 < 0.001, *p*2 = 0.001, p3 = 0.004). All groups differed significantly from the negative control (p = 0.005 for group II, *p* < 0.001 for both groups III and IV). (Table 4; Fig. 3D).

## Discussion

The present study provides a direct comparative evaluation of hDPSCs-EVs and hBP-EVs on the biological behavior of hDPSCs in vitro. The findings demonstrate that both EV sources enhance proliferation and osteo-odontogenic differentiation; however, hDPSCs-EVs exerted significantly superior effects across all functional assays. These results are consistent with the growing body of evidence supporting the regenerative potential of stem cell-derived EVs in dentin–pulp complex repair [1, 23, 24].

The regenerative capacity of the dentin–pulp complex depends on the recruitment, proliferation, and differentiation of endogenous hDPSCs [25] While stem cell transplantation has been widely investigated, concerns regarding tumorigenicity, immunogenicity, and regulatory challenges have shifted attention toward cell-free therapeutic strategies [26] EVs are now recognized as key mediators of stem cell paracrine signaling, transferring bioactive proteins, mRNAs, and microRNAs that regulate recipient cell behavior [24, 26] Our findings reinforce this paradigm by demonstrating that EVs supplementation alone significantly enhanced hDPSCs proliferation and differentiation, even in the absence of direct cell transplantation.

The superior performance of hDPSCs-EVs compared with hBP-EVs may be attributed to lineage-specific cargo specialization. Previous studies have demonstrated that DPSCs-derived EVs are enriched in regulatory molecules involved in odontogenic differentiation pathways, including MAPK and AKT/ERK signaling cascades [27] as well as TGF-β/Smad-mediated mechanisms [28] Huang et al [23] reported that DPSCs-EVs act as biomimetic tools capable of directing stem cell differentiation toward odontogenic lineages. Similarly, Qiao et al. [29] demonstrated enhanced mineralization and DSPP upregulation in periodontal ligament stem cells treated with DPSCs-EVs. These findings align with the significantly higher DSPP expression and OCN positivity observed in the present study.

In contrast, plasma-derived EVs originate from heterogeneous systemic sources, including platelets, erythrocytes, and immune cells [30] While plasma EVs have demonstrated angiogenic and immunomodulatory potential [31] their cargo composition may lack the odontogenic specificity necessary for optimal dentin–pulp regeneration. This heterogeneity could explain the comparatively modest effects of hBP-EVs observed in the present study.

The proliferation results further support the regenerative advantage of tissue-specific EVs. hDPSCs-EVs significantly increased metabolic activity at all evaluated time points, particularly by day 5. In contrast, hBP-EVs did not demonstrate a statistically significant proliferative effect at day 1 compared with the control group, suggesting that hBP-EVs may require longer exposure periods before exerting measurable biological activity on hDPSCs. However, significant proliferative enhancement became evident at later time points, indicating a detectable but delayed influence. These findings are consistent with Ji et al. [32], who reported superior proliferative and immunomodulatory properties of DPSCs-EVs compared with bone marrow-derived EVs. Similarly, Jin et al. [27] demonstrated that DPSCs-EVs can activate MAPK-related signaling pathways associated with osteogenic differentiation and cellular proliferation. In the present study, mechanistic signaling pathway analysis was not performed; therefore, direct attribution of the observed biological effects to specific intracellular pathways cannot be conclusively established.

Mineralization assays revealed a clear hierarchy of osteogenic induction: hDPSCs-EVs > hBP-EVs > osteogenic medium alone. Notably, EVs supplementation enhanced mineral deposition beyond standard osteogenic induction conditions. This observation is consistent with Huang et al. [23], who demonstrated that EVs isolated under odontogenic conditions enhance stem cell differentiation and mineralization. Moreover, Qiao et al. [29] reported dose-dependent increases in mineralized nodule formation following DPSCs-EVs treatment, particularly at concentrations comparable to those used in the present study.

DSPP gene expression analysis further substantiated the odontogenic influence of hDPSCs-EVs. DSPP is considered a specific marker of odontoblastic differentiation [33]. The marked upregulation observed in the hDPSCs-EV group aligns with previous reports demonstrating EV-mediated enhancement of odontogenic gene expression [23, 29]. Additionally, immunocytochemical detection of OCN, a late marker of osteogenic maturation, confirmed enhanced mineralization in EVs-treated groups, particularly those receiving hDPSCs-EVs. These findings correspond with those of Jin et al. [27], who reported increased OCN expression following DPSCs-EVs treatment.

Despite these promising findings, several limitations warrant critical consideration. First, EVs dosing was standardized according to protein concentration rather than particle number. Given differences in particle-to-protein ratios between groups, biological effects may have been influenced by cargo density variations. Future investigations should incorporate particle-normalized dosing to ensure more precise comparative analyses in accordance with current EVs research standards [34].

Second, although ultracentrifugation remains a widely accepted isolation method [25] it may co-isolate non-vesicular protein aggregates. While positive EV markers were confirmed via Western blot, additional negative markers would further strengthen adherence to ISEV recommendations [34].

Third, the present study was conducted entirely in vitro. The dentin–pulp microenvironment is influenced by vascularization, immune modulation, and inflammatory mediators [25], none of which are fully replicated in cell culture systems. Therefore, future in vivo and animal studies are essential to determine whether the observed superiority of hDPSCs-EVs persists under physiological conditions and to evaluate their regenerative performance within the complex dentin–pulp microenvironment.

Furthermore, donor variability was limited, as only healthy male donors aged 20–35 years were included. Age- and systemic-condition-related differences in EV cargo composition have been documented in other contexts [31], which may influence regenerative outcomes. Broader donor representation would enhance generalizability.

From a translational perspective, a balance must be considered between biological specificity and clinical feasibility. While hDPSCs-EVs demonstrated superior regenerative effects in vitro, hBP-EVs offer advantages in accessibility, scalability, and minimally invasive procurement [30, 31]. Thus, although tissue-specific EVs appear biologically advantageous, optimization of scalable DPSCs-EVs production systems will be essential for clinical translation.

*In conclusion*, the present findings support the concept that tissue origin significantly influences EVs bioactivity. hDPSCs- EVs demonstrated enhanced proliferative and osteo-odontogenic effects compared with hBP-EVs, highlighting the importance of lineage-specific signaling in regenerative endodontics. Future investigations should incorporate mechanistic pathway analyses, including MAPK/ERK, AKT, and TGF-β/Smad signaling evaluation, in addition to particle-standardized dosing strategies and in vivo validation models, to further clarify the molecular basis and translational potential of DPSCs-derived EVs in dentin–pulp regeneration.

## Data Availability

Study data can be provided by the corresponding author when reasonably requested.
